# Genomewide Variation in an Introgression Line of Rice-*Zizania* Revealed by Whole-Genome re-Sequencing

**DOI:** 10.1371/journal.pone.0074479

**Published:** 2013-09-18

**Authors:** Zhen-Hui Wang, Di Zhang, Yan Bai, Yun-Hong Zhang, Ying Liu, Ying Wu, Xiu-Yun Lin, Jia-Wei Wen, Chun-Ming Xu, Lin-Feng Li, Bao Liu

**Affiliations:** 1 Key Laboratory of Molecular Epigenetics of Ministry of Education (MOE) and Institute of Genetics and Cytology, Northeast Normal University, Changchun, China; 2 Faculty of Agronomy, Jilin Agricultural University, Changchun, China; 3 Jilin Academy of Agricultural Sciences, Changchun, China; Ben-Gurion University, Israel

## Abstract

**Background:**

Hybridization between genetically diverged organisms is known as an important avenue that drives plant genome evolution. The possible outcomes of hybridization would be the occurrences of genetic instabilities in the resultant hybrids. It remained under-investigated however whether pollination by alien pollens of a closely related but sexually "incompatible" species could evoke genomic changes and to what extent it may result in phenotypic novelties in the derived progenies.

**Methodology/Principal Findings:**

In this study, we have re-sequenced the genomes of *Oryza sativa* ssp. *japonica* cv. Matsumae and one of its derived introgressant RZ35 that was obtained from an introgressive hybridization between Matsumae and *Zizania* *latifolia* Griseb. in general, 131 millions 90 base pair (bp) paired-end reads were generated which covered 13.2 and 21.9 folds of the Matsumae and RZ35 genomes, respectively. Relative to Matsumae, a total of 41,724 homozygous single nucleotide polymorphisms (SNPs) and 17,839 homozygous insertions/deletions (indels) were identified in RZ35, of which 3,797 SNPs were nonsynonymous mutations. Furthermore, rampant mobilization of transposable elements (TEs) was found in the RZ35 genome. The results of pathogen inoculation revealed that RZ35 exhibited enhanced resistance to blast relative to Matsumae. Notably, one nonsynonymous mutation was found in the known blast resistance gene *Pid3/Pi25* and real-time quantitative (q) RT-PCR analysis revealed constitutive up-regulation of its expression, suggesting both altered function and expression of *Pid3/Pi25* may be responsible for the enhanced resistance to rice blast by RZ35.

**Conclusions/Significance:**

Our results demonstrate that introgressive hybridization by *Zizania* has provoked genomewide, extensive genomic changes in the rice genome, and some of which have resulted in important phenotypic novelties. These findings suggest that introgressive hybridization by alien pollens of even a sexually incompatible species may represent a potent means to generate novel genetic diversities, and which may have played relevant roles in plant evolution and can be manipulated for crop improvements.

## Introduction

It has been established that hybridization between genetically differentiated species or ecotypes may induce structural genomic changes (e.g., rearrangement) in the hybrids [1-7]. To address the genetic consequences of inter-specific hybridization, a larger number of studies have investigated the genetic variation patterns and molecular modes of the resultant hybrids. For example, Zou et al. [8] constructed a population of recombinant inbred lines of the new introgressed type of *Brassica napus* (A^r^A^r^C^n^C^n^) through two rounds of inter-specific hybridization between *B. napus* (A^n^A^n^C^n^C^n^) and *B* *. rapa* (A^r^A^r^). As expected, a remarkable range of novel genomic alterations occurred immediately after inter-specific hybridization and some of these genetic changes have profound effects on the yield-related traits in the introgressed *B. napus*. In addition, hybridization may also constitute a "genome shock" and lead to widespread activation of otherwise quiescent transposable elements (TEs) [9]. For instance, Yasuda et al. [10] have examined the mobilizations of *mPing* in 17 introgression rice lines that were derived from the introgressive hybridization between *Oryza sativa* and *O* *. glaberrima* , and illustrated that introgression of genomic DNA from the wild species provoked transpositional activation of *mPing*.

To date, accumulated evidence showing that extensive genetic variations in resultant hybrids has led to the proposition that hybridization can rapidly generate novel phenotypes and hence contribute to the rapid origin of complex adaptations and evolutionary novelties, and ultimately increases overall biodiversity [11-14]. However, diploid (homoploid) hybrids of different species often face a seemingly intangible enigma, that is, the different parental chromosomes are unable to pair correctly during meiosis, and hence lead to sterility [15,16]. This problem can be resolved by whole genome doubling leading to allopolyploid formation, and this may explain why hybridization and polyploidy are often associated in plants [17,18]. However, a great majority of the allopolyploidization events would be met with extinction or an evolutionary dead-end [19] due to "minority cytotype disadvantage" among other problems [20,21].

Nevertheless, the pollination by alien species’ pollens may occur frequently in nature because congeneric species usually display parapatric or sympatric distributions and share common or similar pollinators [22-26]. Apart from *bona fide* hybridization, the germinating alien pollens may constitute a "biological stress" to the recipient stigma genome due to its mechanistic resemblance to pathogen attack wherein the foreign DNA usually does not integrate into the host genome, yet its interaction with the host could provoke genetic and epigenetic instabilities of the host genome [27-30]. Therefore, in contrast to the hybridization and polyploidy, alien pollen-induced variations may represent an underlying mechanism that can also contribute to plant genome evolution.

To address the possible genomic impact of alien pollens on recipient plant genomes, three introgressant lines (RZ1, RZ2 and RZ35) were generated via a "repeated pollination" method [31]. In brief, the panicles of cv. Matusmae were emasculated and pollinated with the pollens of *Z* *. latifolia* . Forty to fifty hours later, the panicles of Matusmae were pollinated for the second time by its own pollens. All of the introgression lines were derived from a single "F_1_" plant and then propagated by strict selfing. In a series of previous studies, we have shown that although a small amount of chromatin of *Z* *. latifolia* (< 0.1%) have been integrated into the rice ssp. *japonica* cv. Matsumae genome by the aforementioned method, these introgressants not only showed extensive *de novo* genetic changes but also exhibited morphological and physiological novelties [30-36]. These studies have provided compelling albeit circumstantial evidence showing that alien pollens can indeed provoke genetic and epigenetic instabilities of a recipient plant genome. Nonetheless, all previous studies are based on traditional molecular marker analysis, and hence, the analyzed loci are limited and potentially biased.

To gain further insights into the genomic effects of alien introgression from an unbiased, genomewide perspective, we have re-sequenced the genomes of a typical introgressant RZ35 along with its rice parental cultivar (Matsumae) using the next generation sequencing technology (Illumina HiSeq 2000). The specific aims of this investigation were: (1) to unravel the types and spectra of the induced genomic changes in the introgression line RZ35; (2) to investigate whether the induced genomic changes occurred randomly across the genome or in a locus-specific fashion in RZ35; and (3) to address to what extent the genomic variations might be linked to phenotypic novelties manifested in RZ35.

## Materials and Methods

### Plant materials, DNA extraction and whole genome re-sequencing

A stabilized introgression line RZ35 (selfed for > 20 generations) derived from introgressive hybridization between rice (*Oryza sativa* ssp. *japonica* cv. Matsumae) and a sexually incompatible wild species of the tribe *Oryzeae*, *Zizania* *latifolia* , was produced by us [31,33]. Genomic DNAs were extracted from leaf tissue of RZ35 and its recipient rice parent cv. Matsumae using a modified CTAB method [37], and purified by phenol extractions. Quality of the DNA samples was determined by a ND-1000 NanoDrop spectrophotometer (Eppendorf, Germany). Approximately 350 µg of genomic DNA of each line was used for Illumina resequencing by the HiSeq 2000 sequencer.

### Pathogen inoculation

One-month-old seedling-plants of Matsumae and RZ35 (at the 4th to 5th leaf stage) were inoculated with blast fungus (*Magnaporthe grisea* (Hebert) Barr) according to previously reported procedures [38]. The inoculated seedlings were placed in an auto-controlled incubator for 24 hours in darkness under the conditions of 28°C and >95% relative humidity.

### Identification of genomic changes in RZ35

For all data sets, only reads ≥ 90 base pair (bp) in length were evaluated. Alignment of Matsumae raw data were initially screened against the Nipponbare reference genome (MSU7.0 http://rice.plantbiology.msu.edu/index.shtml) using Burrows-Wheeler Aligner [39]. Those reads that showed either multiple mapping to the reference genome of Nipponbare or have mapping quality scores less than 30 (Phred scale) were removed from the assembled data. Thereafter, we employed Perl scripts to produce a theoretical "Matsumae reference" genome by including the sequences containing variations in comparison with Nipponbare. The remaining reads of RZ35 were then aligned to assembled Matsumae reference genome to detect homozygous SNPs, INDELs using SAMtools (v0.1.5c). In order to minimize the false-positive rates of SNPs and INDELs, the filter method was applied with the following parameters: Phred score ≥ 30, coverage ≥ 10 and ≤ 100.

### Distribution patterns of genomic changes and identification of mutated genes

To infer the genome-wide pattern of variations and identify the HMRs in the RZ35 genome relative to that of Matsumae, a sliding window with 100 kilo base pair (kb) interval was applied to calculate polymorphisms for each chromosome using Perl scripts and the figures were generated by ggplot2 package in software R. Consequently, the locations of HMRs on each chromosome of RZ35 were compared with the domestication-related regions of rice that were identified previously [40]. In addition, to estimate the base substitution rates, we calculated the ratio of transition (Ts) and transversion (Tv) for each chromosome of RZ35. Furthermore, we also performed a genome scan to identify the positions of these genomic changes. Specifically, the Generic Feature Format Version 3 (GFF3) files of annotated Nipponbare reference were summarized and formatted to a two-dimensional array file that includes exons, introns and ATG locations of each gene. Then, the locations of each genomic variation were determined by calculating the distance between variations and ATG start codon. Finally, the nonsynonymous mutations were identified through the comparisons of protein sequences between RZ35 and Matsumae. The protein sequences of these mutated genes were retrieved and subsequently subjected to Kyoto Encyclopedia of Genes and Genomes (KEGG) to assign their positions in each pathway [41]

### Identification and characterization of transposable elements (TEs) mobilization

Our previous studies have identified transpositional activation or mobilization of several TEs in the rice- *Zizania* introgression lines including RZ35 [30,33,34]. To further investigate if mobilization of additional TEs or mobilization sites of previously identified TEs in the RZ35 genome, we applied a PEM detection strategy (Figure S1) as employed previously by Sabot et al. [42]. In brief, the raw data of Matsumae and RZ35 were aligned against the Nipponbare reference genome (MSU7.0 http://rice.plantbiology.msu.edu/ index. shtml) using bowtie software [43]. Then, all of the non-matching reads were searched against the known sequences of TEs (such as *Tos17*, *Tos19* and *mPing* etc.) using software bowtie again. These reads that showed high similarities to TEs were selected, and the remaining reads were subsequently BLAST search against the Nipponbare reference genome. The chromosome positions of these non-matching reads were subjected to generate a list that contains the chromosomic regions, inserted element and paired ends of candidate TEs. Those candidates that showed a clear pattern of paired-end mapping on the chromosomal region and transposable element were regarded as mobilization of TEs. To further verify authenticity of the identified TE mobilizations in the RZ35 genome, PCR primers were designed targeting junctions of a set of selected TE insertions using Primer 5 (http://www.premierb iosoft.com) to perform locus-specific amplifications. The primer sequences used in this study were listed in the Table S1. The PCR reaction conditions are 2 min at 94°C; 35 cycles of 45 sec at 94°C, 45 sec at 58°C, 1 min at 72°C; a final extension for 8 min at 55°C. The PCR products were visualized by ethidium bromide staining after electrophoresis through 2% agarose gels.

### RNA isolation and real-time qRT-PCR analysis for blast disease-resistance genes

Total RNA was isolated from young seedling at the same developmental stage (at the 3^th^-4^th^ leaf-stage) from RZ35 and Matsumae with Trizol Reagent (Invitrogen) according to the manufacturer’s protocol. The RNA was treated with DNase I (Invitrogen) to eliminate possible genomic DNA contamination before being reverse transcribed with the SuperScript Rnase H-Reverse Transcriptase (Invitrogen). The results of pathogen inoculation experiment revealed that RZ35 exhibited enhanced resistance to rice blast. Therefore, to analyze possible alteration in constitutive expression of 10 previously identified blast disease-resistance genes, gene-specific qRT-PCR primers were designed using Primer 5 (Table S2). All of these identified genes were retrieved from the China Rice Data Center (http://www.ricedata.cn/index.htm). The qRT-PCR reactions were conducted with an initial step at 95°C for 1 min followed by 45 cycles of 5 sec at 95°C, 10 sec at 60°C and 30 sec at 72°C. The relative expression of the analyzed genes was calculated using the Δ Ct method.

## Results

### Mapping of Illumina reads and detection of genomewide genetic changes in RZ35

In this study, a survey of the genome re-sequencing data for both Matsumae and RZ35 yielded 131 million 90 base pair (bp) paired-end reads in total, and of which 123 million reads were successfully mapped to the Nipponbare reference genome (MSU7.0 http://rice.plantbiology.msu.edu/index.shtml). Notably, 92 million reads aligned uniquely to the reference genome, whereas about 30 million reads showed multiple locations (Table 1). All reads together yielded 13.2x and 21.9x coverage of the Matsumae and RZ35 genomes, respectively. Next, through alignment to the Matsumae genome as a reference, a total of 59,563 homozygous genetic changes were identified in RZ35, which included 41,724 single nucleotide polymorphisms (SNPs), 8,418 insertions and 9,421 deletions (Table 2). All of these DNA sequences have been submitted to GenBank under the accession numbers of SRA072822.

**Table 1:** Number of GA reads and coverage of the genome.

	Matsumae	RZ35
FASTQ	24646673	40697827
Coverage	13.2	21.9
Unique map	34584615	57860050
Mutltiple map	11356645	18892937
Unmapped	3352086	4642667

**Table 2:** Polymorphism in genomic DNA observed in the RZ35 compared with Matsumae.

Chromosome	Chr. length (bp)	No. SNPs	SNP/100kb	No. insert.	Insert. /100kb	No. del.	Del. / 100kb	Ts:Tv
1	43268879	4217	9.75	1046	2.42	1215	2.81	2.33
2	35930321	2534	7.05	697	1.94	794	2.21	2.40
3	36406629	1485	4.08	478	1.31	490	1.35	2.87
4	35278165	3595	10.19	834	2.36	872	2.47	1.84
5	29894729	1259	4.21	340	1.14	373	1.25	2.84
6	31246729	6265	20.05	1119	3.58	1246	3.99	2.49
7	29696569	2000	6.73	591	1.99	669	2.25	2.32
8	28439248	1708	6.01	462	1.62	537	1.89	2.42
9	23011178	2729	11.86	510	2.22	594	2.58	2.56
10	23134699	9478	40.97	1070	4.63	1220	5.27	2.38
11	28512606	3844	13.48	737	2.58	821	2.88	2.47
12	27497154	2610	9.49	534	1.94	590	2.15	2.33
Total/Ave.	372316906	41724	11.99	8418	2.26	9421	2.53	2.38

### Nucleotide substitution rates and variation patterns of genomic changes in RZ35

**Figure 1:**
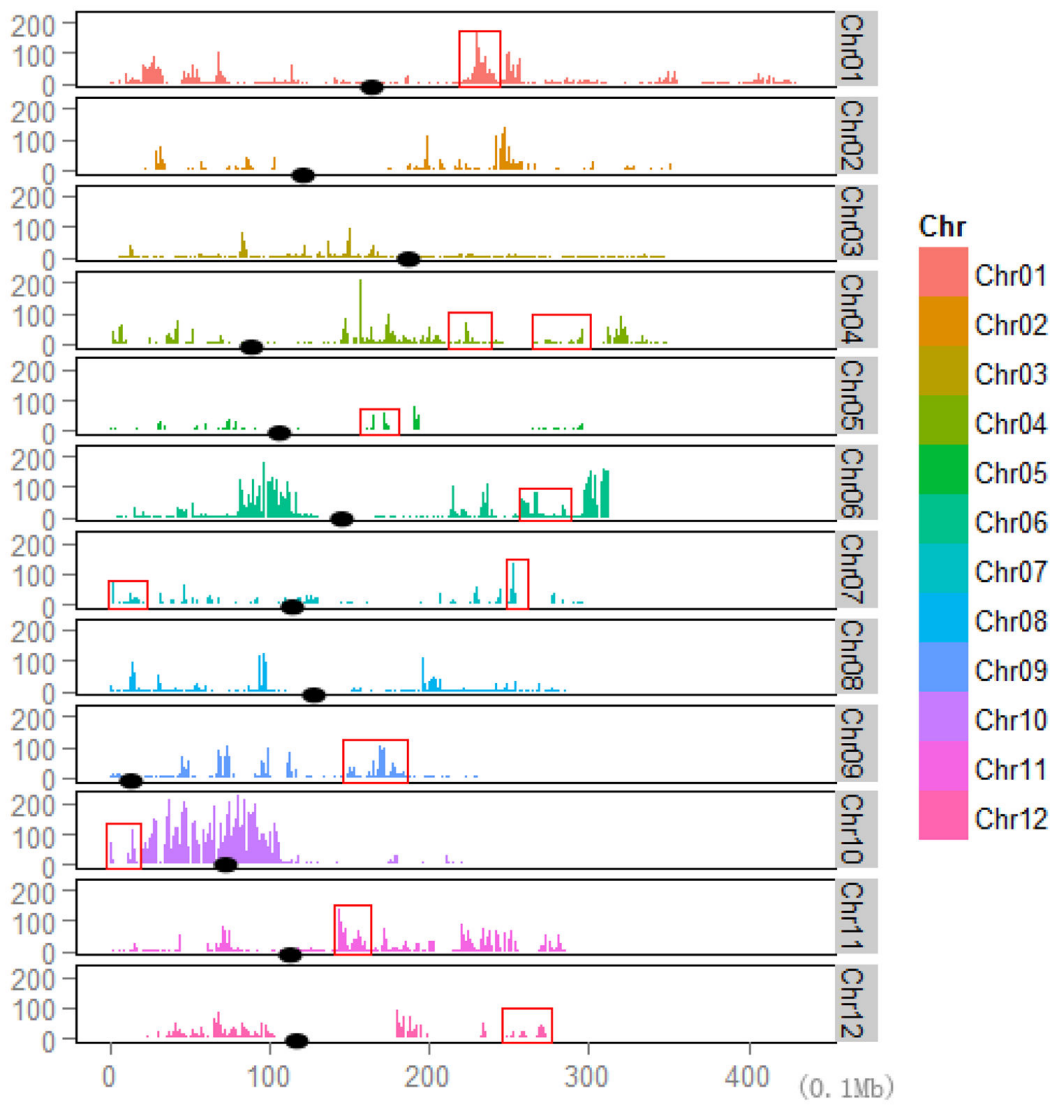
Distribution of SNPs within 100 kb windows across each chromosome (chr). For a change of color denotes different chromosomes. The x axis stands for each chromosome divided by 0.1 Mb. The y axis stands for the number of SNPs. The black solid ellipse stands for the centromere. The red frames stand for the domestication related regions which were found in rice ssp. *japonica*.

Estimation of nucleotide substitution rates was obtained through the comparison between assembled genomes of Matsumae and RZ35. In general, a high nucleotide substitution rate (5.6x10^-6^ per nucleotide site) was found in RZ35 and these genomic variations showed an uneven distribution across each of the 12 rice chromosomes (Figure 1). The average densities of nucleotide polymorphisms were determined on a per 100 kb basis across each chromosome, and this gave rise to an average of 11.99 SNPs, 2.26 insertions and 2.53 deletions if all chromosomes were considered together in RZ35 relative to Matsumae (Table 2). In addition, according to the analysis of base substitution model, two most frequent types of substitution (G/A and C/T) were observed in the RZ35 genome, leading to a relatively higher substitution rate of Ts than Tv (Ts:Tv = 2.38) (Figure S2 and Table 2). Notably, several hyper-mutation regions (HMRs) were identified in the genome of RZ35 (Figure 1), and most of these HMRs occurred in the euchromatin regions of each chromosome, except in chromosome 10 where HMRs were located near the centromere. In addition, chromosome 10 also exhibited the highest SNP and insertions/deletions (INDELs) mutations among all 12 chromosomes (Table 2). Interestingly, it is noted that 11 of these HMRs were associated with domestication (Figure 1). Through search against the available database of *O. sativa*, we found that a small amount of these genomic changes were shared with other rice cultivars (data not shown). It indicates that the introgressive hybridization has triggered novel genetic changes in the RZ35 genome.

### Classification of SNPs within mutated genes in RZ35

**Figure 2:**
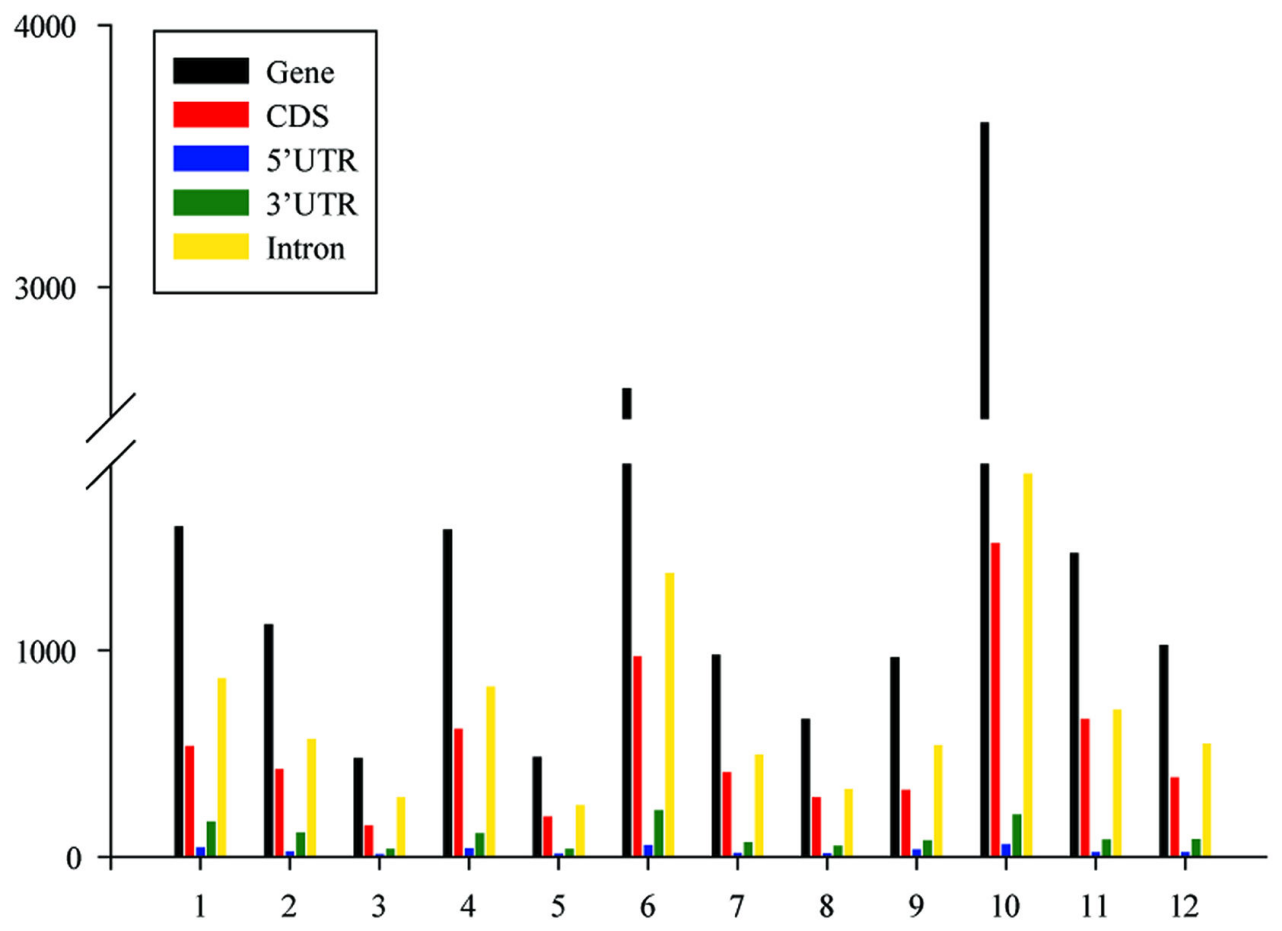
Diagrams showing distribution of homozygous single nucleotide polymorphisms (SNPs) in genes according to each chromosome. The x axis numbers stand for the each chromosome. The y axis numbers stand for the numbers of the homozygous SNPs. The different colors stand for the different components of the genes.

According to the analyses of global patterns of genomic variation in RZ35, a total of 16,571 SNPs were identified in genes, of which 6,433 were located in coding regions and 8,596 were found in introns (Figure 2). Importantly, 3,797 nonsynonymous mutations were detected in the RZ35 genome and which occurred in 2,250 genes (Additional File S1). Notably, a large proportion of these nonsynonymous mutations were found in the transpose-coding genes of transposons and retrotransposons, and NBS-LRR protein-coding genes, suggesting these amino acid mutations may lead to the previously characterized phenotypic novelties of RZ35 [31,44].

**Figure 3:**
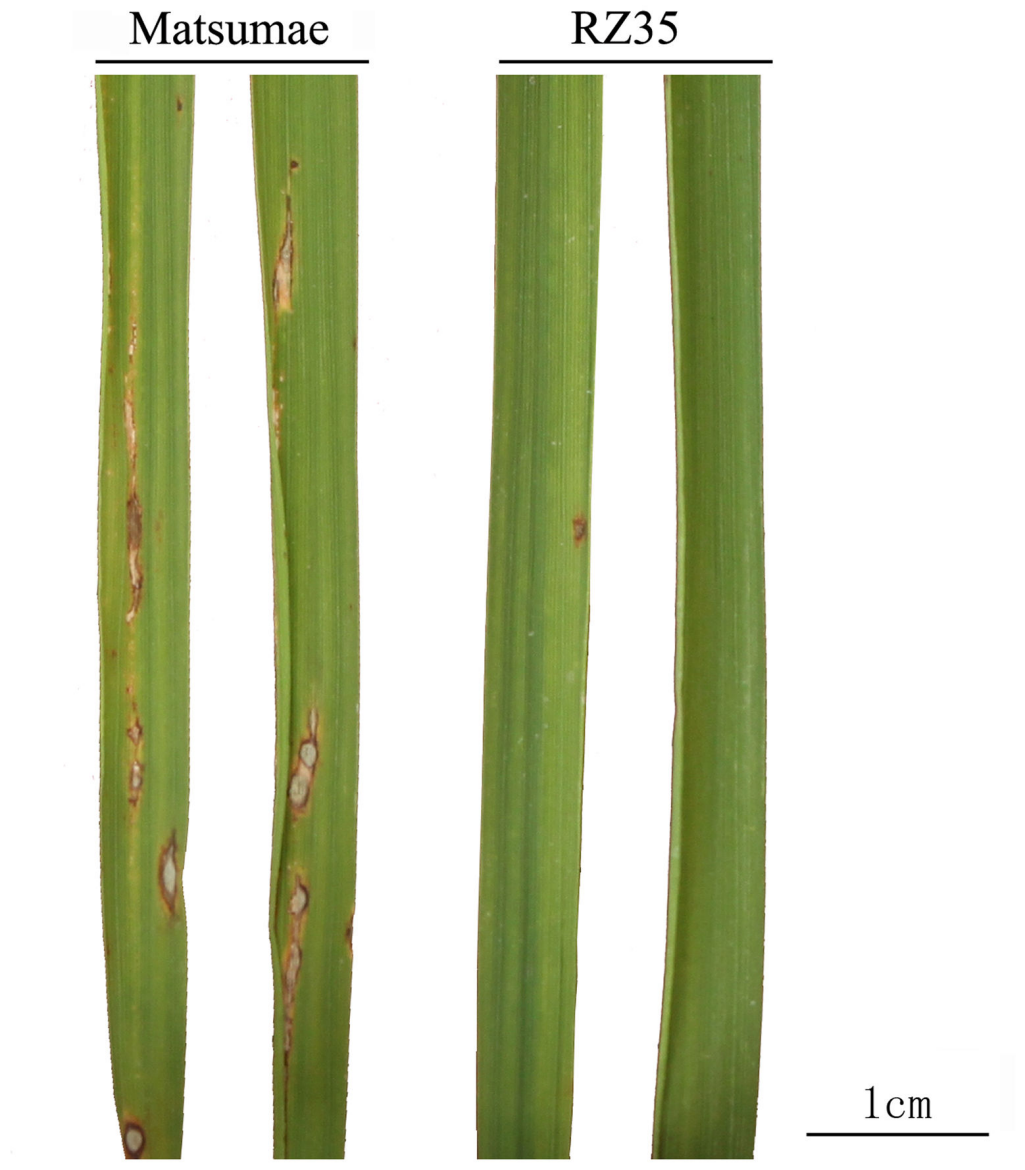
Comparisons of representative phenotype of Matsumae and RZ35. This phenotype was generated at a 7-day period after *Magnaporthe grisea* infection and the scale bar indicates 1cm.

**Figure 4:**
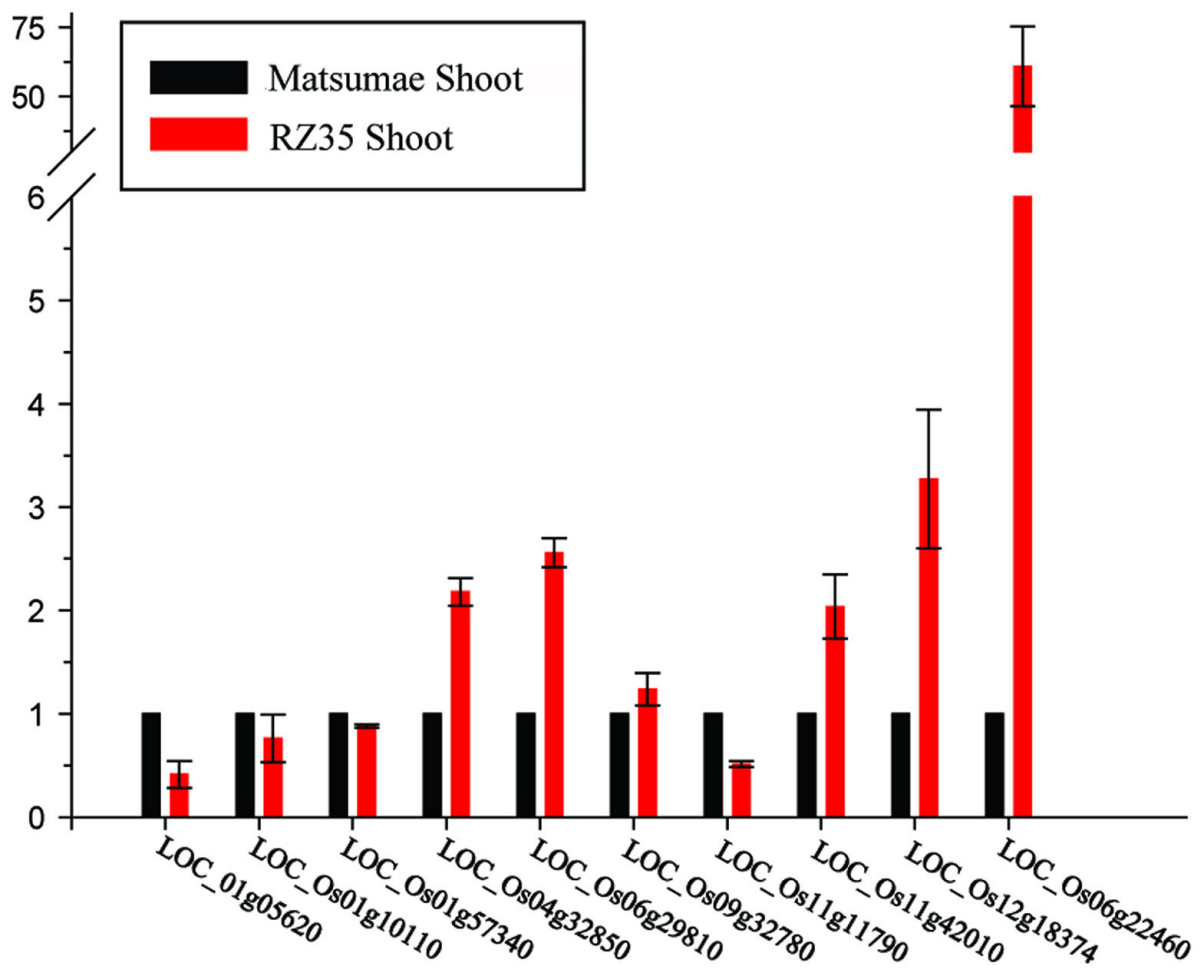
Measurements of expression of the 10 major blast-resistance genes in the leaf tissue taken from of Matsumae and RZ35, measured by real time qRT-PCR.

As it is known from our previous studies that RZ35 has a significantly enhanced resistance to the blast disease relative to its rice parental cultivar Matsumae (e.g., Figure 3) [31], it is of apparent interest to investigate whether the known and putative blast-resistance genes identified in the rice genome are mutated in RZ5 relative to Matsumae. Indeed, we found that a total of 94 nonsynonymous mutations occurred in 40 NBS-LRR disease resistance genes, and one nonsynonymous mutation occurred in a known blast-resistance gene *Pid3/Pi25* (LOC_Os06g22460) (Figure S3). Therefore, we performed qRT-PCR assay for expression of *Pid3/Pi25* along with other nine non-mutated blast-resistance genes to test whether there was differential constitutive expression between RZ35 and Matsumae for these genes. We found that five of these nine genes showed up-regulation in RZ35 relative to Matsumae, and *Pid3/Pi25* showed the highest up-regulation (Figure 4). These findings implied that *Pid3/Pi25* might be responsible for the blast-resistance phenotype of RZ35. Furthermore, identification of the positions of these mutated genes revealed that a total of 1,244 genes were located in 54 pathways and some mutated genes participated in the glycolysis, fatty acid, phenylpropanoid biosynthesis and fatty acid, purine, pyrimidine metabolism pathways (Figure S4-9). Importantly, most of these mutated genes within these pathways were located in the HMRs, suggesting they may confer other phenotypic changes manifested in RZ35 [31,33,45,46].

### Identification and verification of mobilizations of transposable elements (TEs) in RZ35

**Figure 5:**
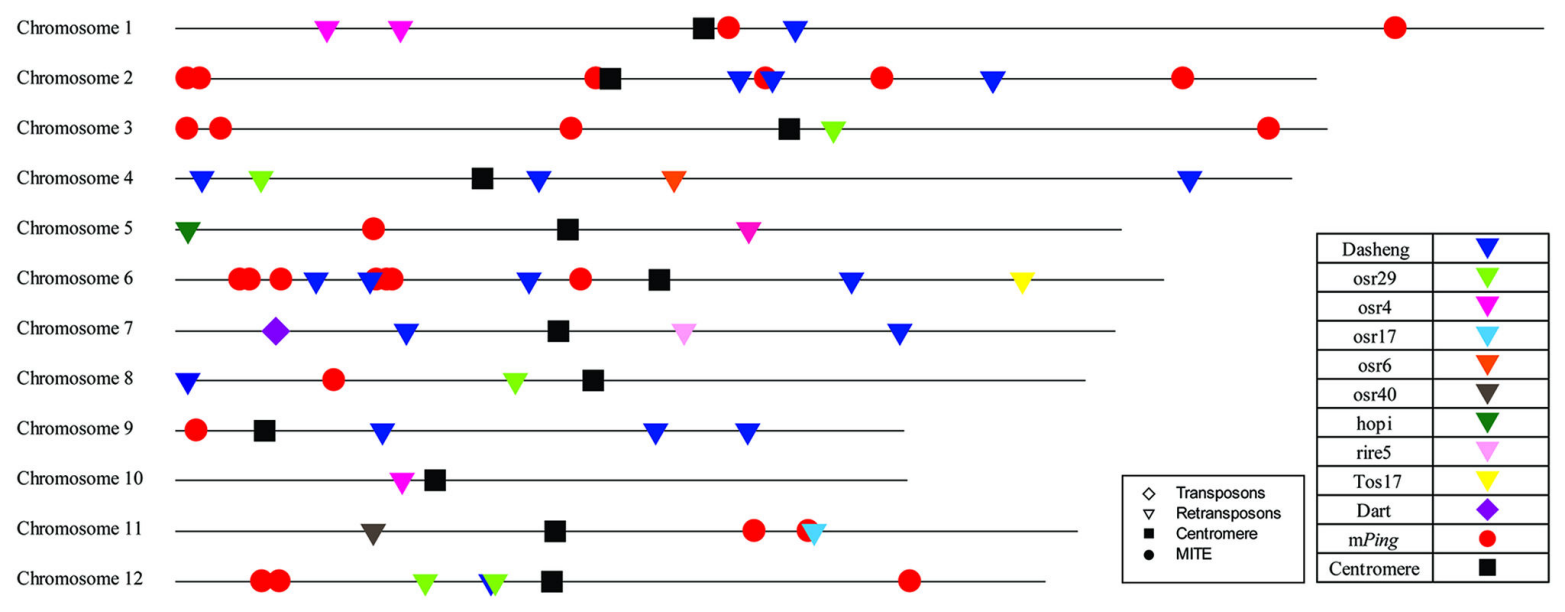
*In silico* mapping of the 63 *de novo* TE insertions detected using PEM method on each chromosome. Arrows represent LTR retrotransposons, diamonds and red dots represent Dart and *mPing*, respectively.

Through the paired-end mapping (PEM) approach, genome-wide transpositional reactivations of several TEs were detected in the RZ35 genome (Figure 5). Specifically, a total of 63 mobilization events by 11 TE families were identified in RZ35, of which 28 and 34 new TE insertions were caused by *mPing* and LTR retrotransposons, respectively (Table 3). It should be noted that most of the mobilization events appeared to occur in gene-rich regions and 18 new insertions were located in the intron and upstream of transcription start (< 1 kb) (Table S3). Of these mobilization events of TEs in RZ35, 11 insertion events representing the 11 TE families were selected and independently tested by locus-specific PCR amplifications anchoring the insertion junctions, and all these mobilization events were validated by this independent analysis (Figure S10).

**Table 3:** The analyzed TEs and their New TE insertions identified in RZ35 and their original copy numbers in Matsumae based on PEM mapping.

TE name	TE type	No. new TE insertions in RZ35	No. original TE copies in Matsumae
*Dasheng*	LTR retrotransposon	19	289
*osr29*	LTR retrotransposon	5	74
*osr4*	LTR retrotransposon	4	29
*osr17*	LTR retrotransposon	1	13
*osr6*	LTR retrotransposon	1	5
*osr40*	LTR retrotransposon	1	120
*Hopi*	LTR retrotransposon	1	270
*Rire5*	LTR retrotransposon	1	18
*Tos17*	LTR retrotransposon	1	4
*Dart*	DNA Transposon	1	54
*mPing*	MITE	28	55
Total		63	

## Discussion

### Distribution patterns of the introgressive hybridization-induced genomic changes

It has been documented by an array of studies that introgressive hybridization can indeed evoke extensive and genomewide genetic variations in the resultant hybrids [4,47-55]. In addition to the *bona fide* hybridization, our previous studies have demonstrated that pollination by alien pollens can also induce *de novo* genomic changes in the rice- *Zizania* introgression lines [30,31,33-35]. Nevertheless, because these studies are based on molecular markers sampling limited loci, it remained unclear the extent of the induced genomic changes and their distribution patterns on a genomewide scale. Here, based on deep-coverage whole genome re-sequencing, we show that genomewide genomic changes occurred in a typical introgression line of rice- *Zizania* , RZ35. Analysis of base substitutions indicated that the frequencies of nucleotide transition are significantly higher than those of transversion. This nucleotide substitution bias is in broad consistence with previous studies that also showed higher frequencies of transition than transversion among natural species [56-59], thus corroborating that the genomic variations in RZ35 were actually triggered by introgression of *Z* *. latifolia* rather than some unknown stochastic factors during the experimental process.

Notably, our results have demonstrated that the nucleotide variations tended to localize in clusters in a given chromosome rather than being randomly distributed. Although such non-randomness with respect to the accumulation of SNPs were also found previously among rice cultivars [60], we note that only a very small amount of the HMRs uncovered in RZ35 is shared by those among the existing rice cultivars, suggesting that novel genetic variations have been induced in rice by the introgressive hybridization with *Zizania* . Rates of nucleotide substitution are known to vary widely across the genome of natural plant species due to the variable efficacy of selection and genetic drift against mutations [61-64]. In fact, previous studies have implicated that nucleotide substitution rates were expected to be accelerated at those genomic regions under natural selection, but will otherwise be determined by genetic drift only in the neutral regions [65]. Consequently, the genome of natural species is a mosaic of discrete segments and each segment may have its own unique history and contributes differentially to the evolution of a species. This hypothesis is also confirmed by our study because several HMRs were identified across each of the 12 chromosomes of RZ35. Specifically, a number of the mutated genes within HMRs were participated in some biologically important pathways. For example, the phenylpropanoid biosynthesis has a range of important functions in plants, including the synthesis of pigments, growth regulators as well as plant toxins, and all of which can enhance plant’s tolerance to biotic and abiotic stresses [45,46]. Similarly, the biosynthesis of glycolysis, fatty acid as well as the metabolism of fatty acid, purine and pyrimidine pathway have also contributed to the abilities of plants in response to environmental stresses [66-68]. Additionally, the pathogen inoculation experiment demonstrated that RZ35 showed enhanced resistance to rice blast and the constitutively unregulated *Pid3/Pi25* may be responsible for the blast-resistance phenotype of RZ35. Although an alternative possibility remains that the blast resistance of RZ35 was introgressed from *Z* *. Latifolia* , we are more in favor of the scenario that the resistance was generated *de novo*, as a consequence of the genomic changes provoked by the introgressive hybridization based on the following arguments: (1) it is known that less than 0.1% of *Zizania* DNA was integrated into the genome of RZ35 [35], and (2) the blast resistance gene *Pid3/Pi25* of RZ35 showed high homology (99%) to rice. These observations allow us to speculate that the induced genomic variations may be at least partly responsible for the observed morphological and physiological novelties of RZ35 in comparison with Matsumae. Interestingly, we also noted that 11 HMRs were located within the domestication-related regions in rice. A previous study showed that these genomic regions may control domestication-related traits and have been selected during the domestication process of rice [40]. Although the exact functions of these domestication-related regions and how they affect the phenotypes of rice remained unclear, our findings implicated that these regions are likely more fragile and hence labile to mutations as a result of introgressive hybridization. Taken together, our findings suggest that the occurring of genetic variations in RZ35 might have been shaped by some underlying modes during or immediately following the introgression process and these induced genomic changes confer beneficial phenotypes to RZ35.

In contrast to the nonrandom distribution pattern of nucleotide changes discussed above, mobilization of TEs appeared to have occurred randomly across the RZ35 genome. Apart from insertional mutagenesis, transpositionally activated TEs are known to have potent effects on genome structure by inducing both ectopic rearrangements and nucleotide changes at or adjacent to TE excision and insertions sites [69]. Indeed, several studies have revealed that a significant proportion of plant genes have been assembled or amplified via the action of TEs, and most genes contain legacies of multiple TE insertions into their promoter regions [70-72]. Our study showing that most of the *de novo* insertion events by the mobilized TEs occurred in gene-rich regions is consistent with the known propensity of these TEs.

### Implications of introgressive hybridization-induced genomic changes in plant genome evolution and crop improvement

Although the roles of hybridization in plant genome evolution is still under debate with opposing views of hybridization as either evolutionary noise [73] or an engine for generating biodiversity [74], it has been generally accepted that introgressive hybridization provides an efficient means for transfer of important genes from the donor to the recipient genome [75]. However, the question as to whether the introgressive hybridization process *per se* may generate *de novo* genetic variations remain poorly studied [76]. Our previous, molecular marker-based studies in several rice– *Zizania* introgression lines have shown that the introgressive hybridization process, even between these two sexually incompatible combination of rice and *Zizania* , can generate extensive *de novo* genetic changes [33,35], which has been further confirmed in this study at the whole genome scale. Another pertinent question is whether the genetic variations generated by this means might be relevant to genome evolution or can be explored for crop improvement. Given that closely related species usually exhibit parapatric or sympatric distributions and often share similar pollinators [22-26], we believe that pollination by alien species’ pollens may represent a frequent occurrence. Therefore, it is possible that genetic variations produced by this kind of introgressive hybridization process may have played a role in genome evolution under natural conditions. In this aspect, the variations produced by this novel means likely share similar biological consequences as those produced by *bona fide* hybridizations [75,77]. With respect to the possible utility of the genomic variations generated by introgressive hybridization, the introgression lines of rice- *Zizania* have provided a compelling positive answer: several of these lines have been actively used in rice breeding programs, and the line studied here, RZ35, alone has been involved in the release of 18 new rice cultivars.

In conclusion, we have shown by whole genome re-sequencing that introgressive hybridization between rice (cv. Matsumae) and a sexually incompatible wild species, *Z* *. latifolia* , has generated genomewide genomic variations, which included SNPs, INDELS and mobilization of TEs. These extensive genomic variations likely underlie genesis of an array of phenotypic and physiological changes in the introgression line. These genomic and phenotypic variations might occur in nature to facilitate genome evolution and can be readily utilized in crop improvements.

## Supporting Information

File S1 **Details of total homozygous SNPs annotation information.** (XLSX)

Figure S1 **Schematic view of the procedure of PEM that is the same as Sabot et al. [42].**
(TIF)

Figure S2 **The matrix of base substitutions.** The SNPs detected were classified as transitions (C/T and G/A) or transversions (C/G, T/A, A/C and G/T) based on nucleotide substitutions. The numbers on the line refer to the SNP amount for this type. The degree of thickness of the lines was calculated by the times of the minimum number.
(TIF)

Figure S3 **The mutation model of the anti-blast genes.** The red vertical lines stand for the SNP mutation site. The red amino acid sequences represent the amino acid site which has a nonanonymous SNP.
(TIF)

Figure S4 **The locations of mutated genes in the Glycolysis/Gluconeogenesis pathway.** These frames that marketed with green color indicate the positions of each mutated gene.
(TIF)

Figure S5 **The locations of mutated genes in the fatty acid Biosynthesis pathway.** These frames that marketed with green color indicate the positions of each mutated gene.
(TIF)

Figure S6 **The locations of mutated genes in the phenylpropanoid Biosynthesis pathway.** These frames that marketed with green color indicate the positions of each mutated gene.
(TIF)

Figure S7 **The locations of mutated genes in the fatty acid Metabolism pathway.** These frames that marketed with green color indicate the positions of each mutated gene.
(TIF)

Figure S8 **The locations of mutated genes in the purine Metabolism pathway.** These frames that marketed with green color indicate the positions of each mutated gene.
(TIF)

Figure S9 **The locations of mutated genes in the pyrimidine Metabolism pathway.** These frames that marketed with green color indicate the positions of each mutated gene.
(TIF)

Figure S10 **Identifications of transposable element (TE) insertions.** The Illumina amplicons mapped are indicated above the genome sequence. (A) And (B) indicate the strategies of primer designing that is the same as Sabot et al. [42]. (C) Ma: represents Marker, M and R donate Matsumae and RZ35, respectively.
(TIF)

Table S1 **The primer sequences for the identification of transposon elements insertion.**
(DOC)

Table S2 **The primers sequences of anti-blast genes used in this study.**
(DOC)

Table S3 **Information of these genes that new TE insertion was found within 1 kb.**
(DOC)
