# Supplementary figures and images for: Genomewide Variation in an Introgression Line of Rice-Zizania Revealed by Whole-Genome re-Sequencing
Source: PLoS One. 2013 Sep 18;8(9):e74479. doi: 10.1371/journal.pone.0074479 (PMC3776793; doi:10.1371/journal.pone.0074479)

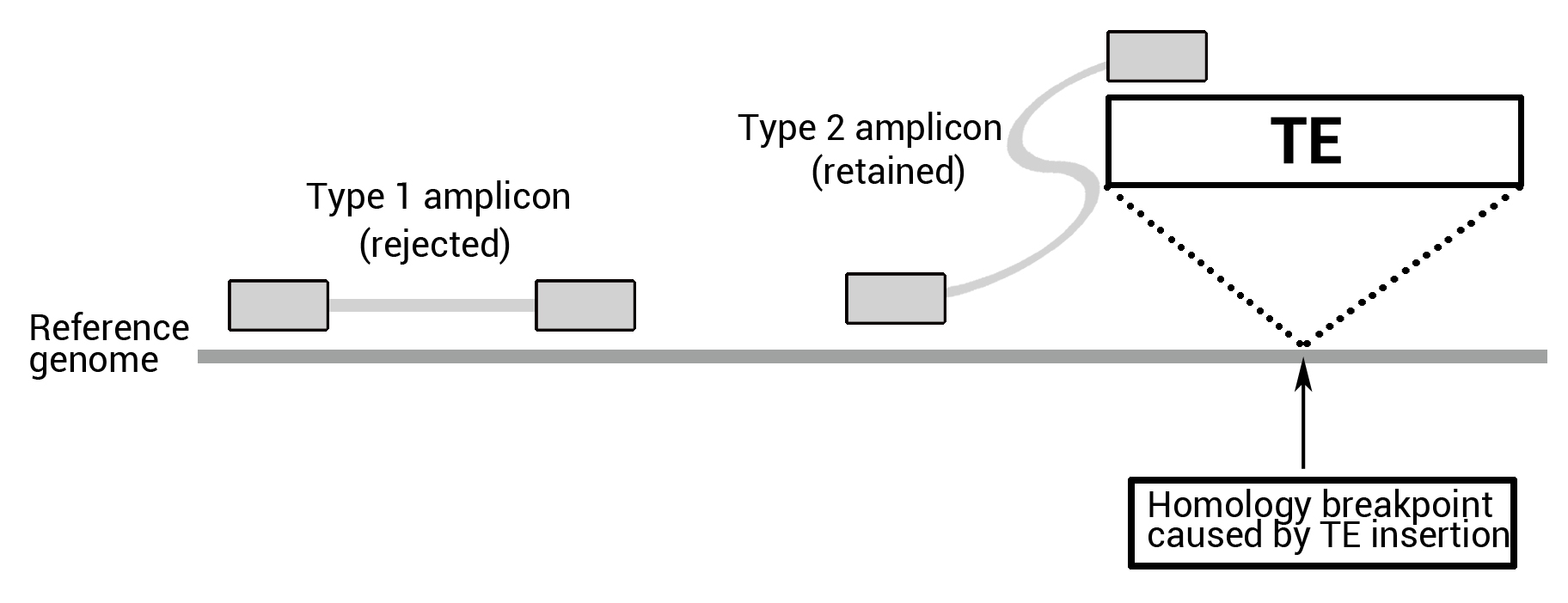

Supplement: Figure S1 — Schematic view of the procedure of PEM that is the same as Sabot et al. [42]. (TIF) [file pone.0074479.s002.tif]

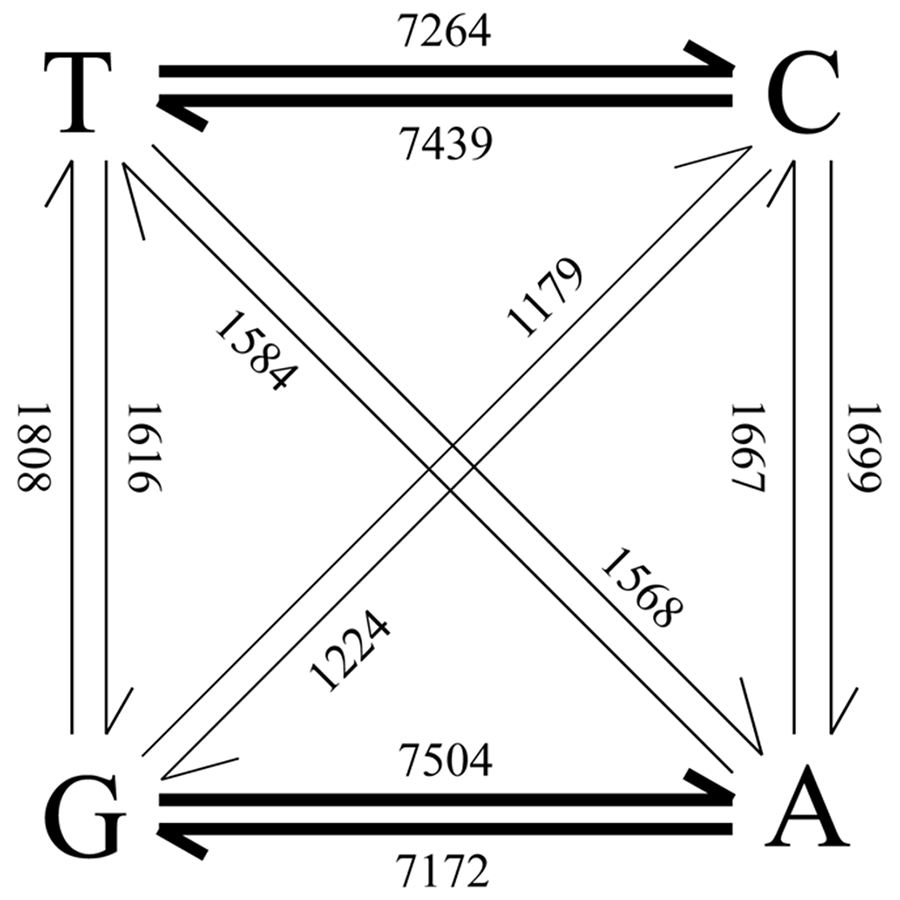

Supplement: Figure S2 — The matrix of base substitutions. The SNPs detected were classified as transitions (C/T and G/A) or transversions (C/G, T/A, A/C and G/T) based on nucleotide substitutions. The numbers on the line refer to the SNP amount for this type. The degree of thickness of the lines was calculated by the times of the minimum number. (TIF) [file pone.0074479.s003.tif]

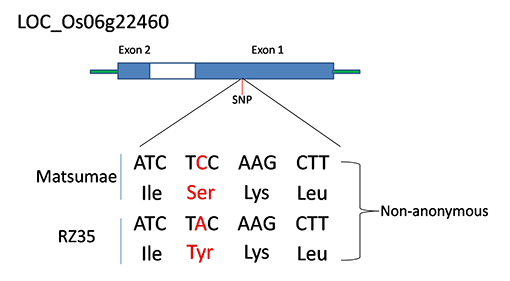

Supplement: Figure S3 — The mutation model of the anti-blast genes. The red vertical lines stand for the SNP mutation site. The red amino acid sequences represent the amino acid site which has a nonanonymous SNP. (TIF) [file pone.0074479.s004.tif]

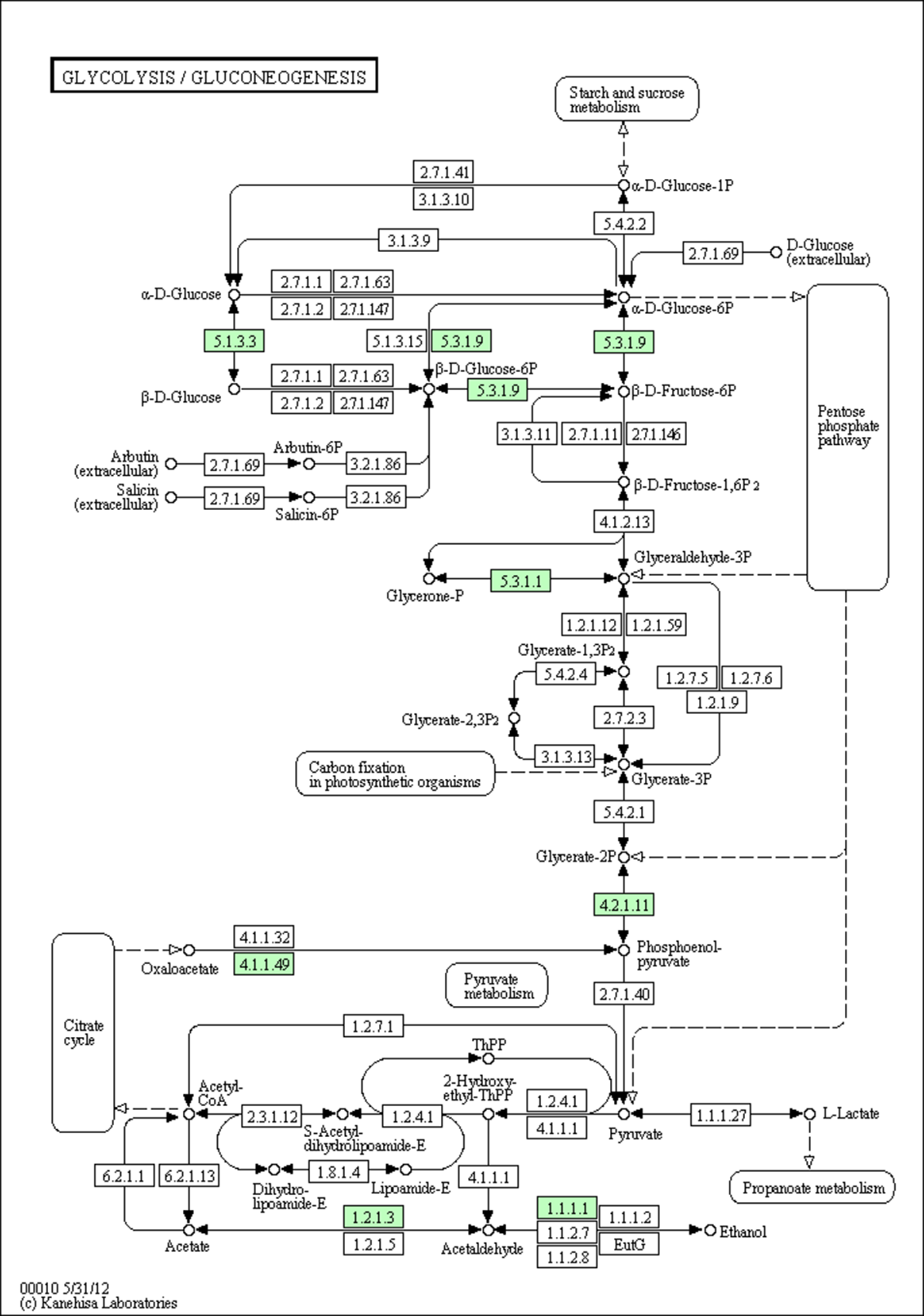

Supplement: Figure S4 — The locations of mutated genes in the Glycolysis/Gluconeogenesis pathway. These frames that marketed with green color indicate the positions of each mutated gene. (TIF) [file pone.0074479.s005.tif]

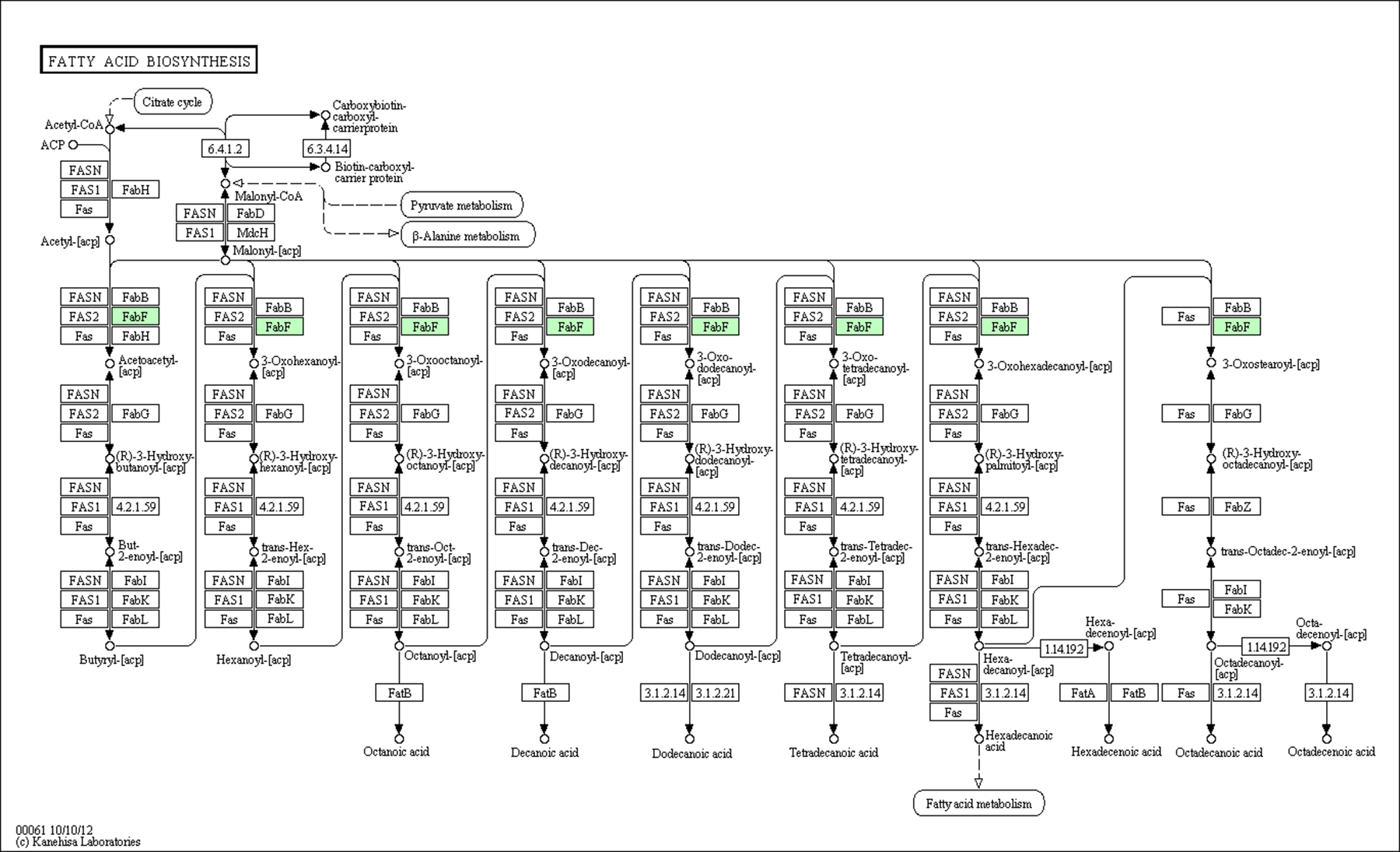

Supplement: Figure S5 — The locations of mutated genes in the fatty acid Biosynthesis pathway. These frames that marketed with green color indicate the positions of each mutated gene. (TIF) [file pone.0074479.s006.tif]

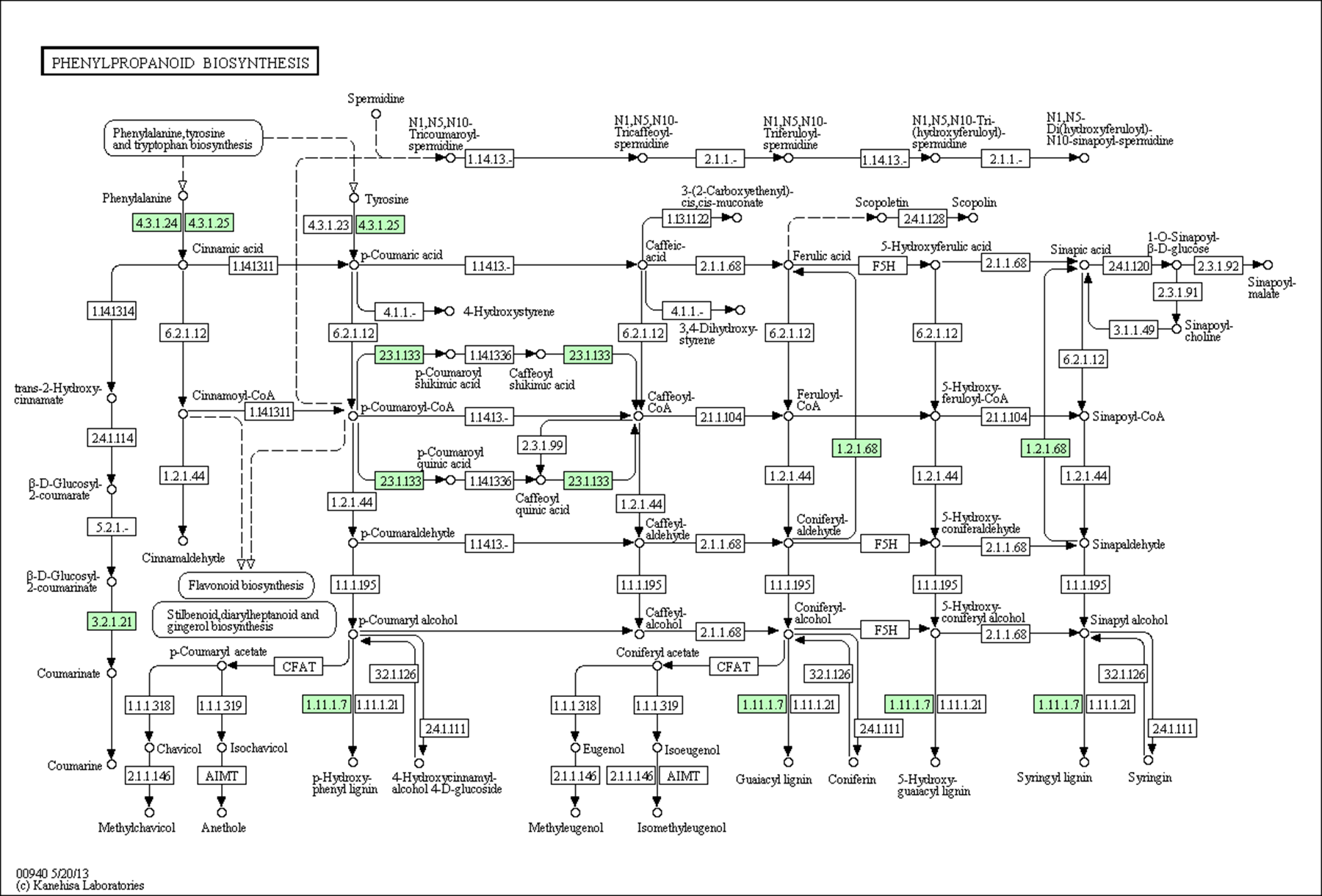

Supplement: Figure S6 — The locations of mutated genes in the phenylpropanoid Biosynthesis pathway. These frames that marketed with green color indicate the positions of each mutated gene. (TIF) [file pone.0074479.s007.tif]

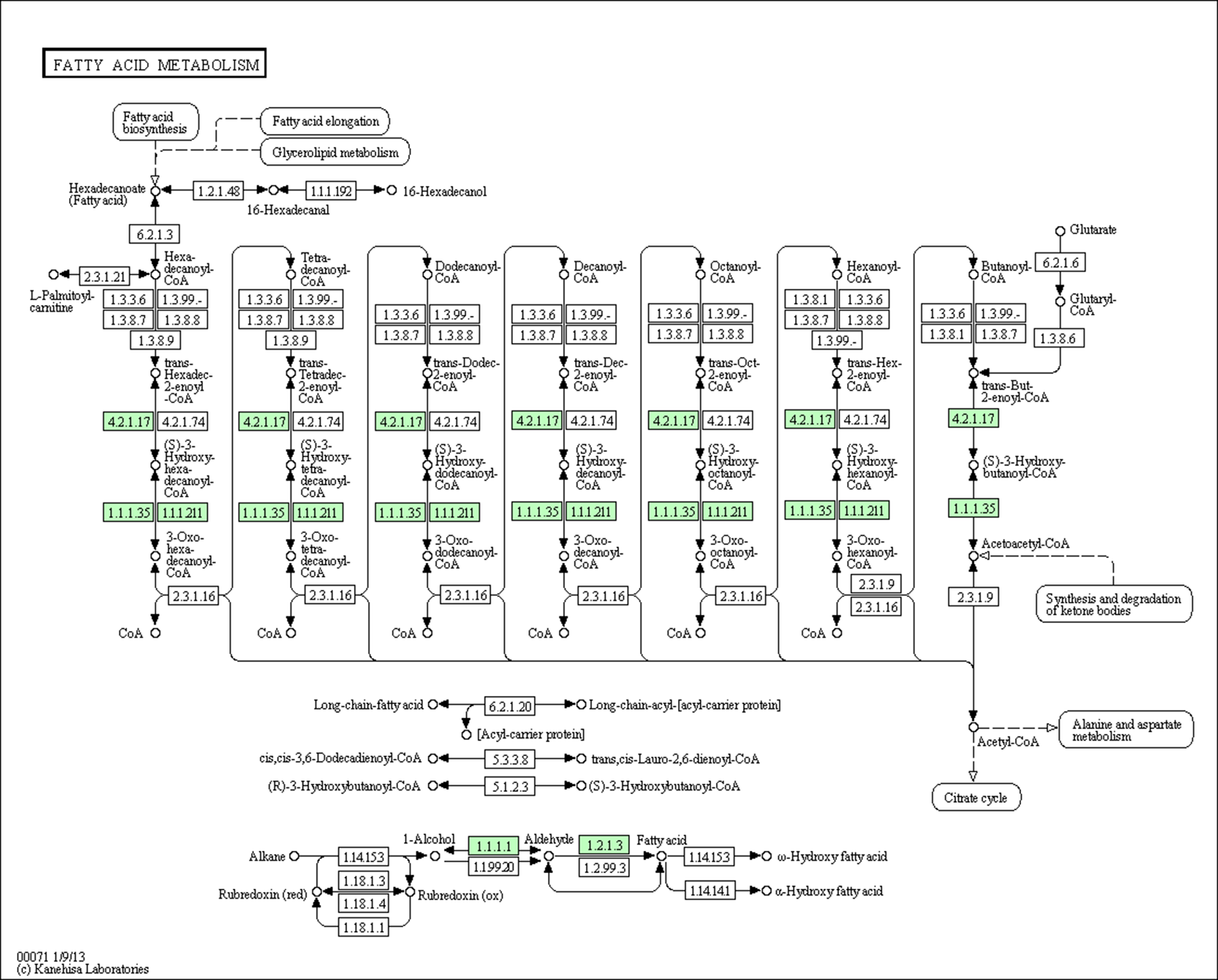

Supplement: Figure S7 — The locations of mutated genes in the fatty acid Metabolism pathway. These frames that marketed with green color indicate the positions of each mutated gene. (TIF) [file pone.0074479.s008.tif]

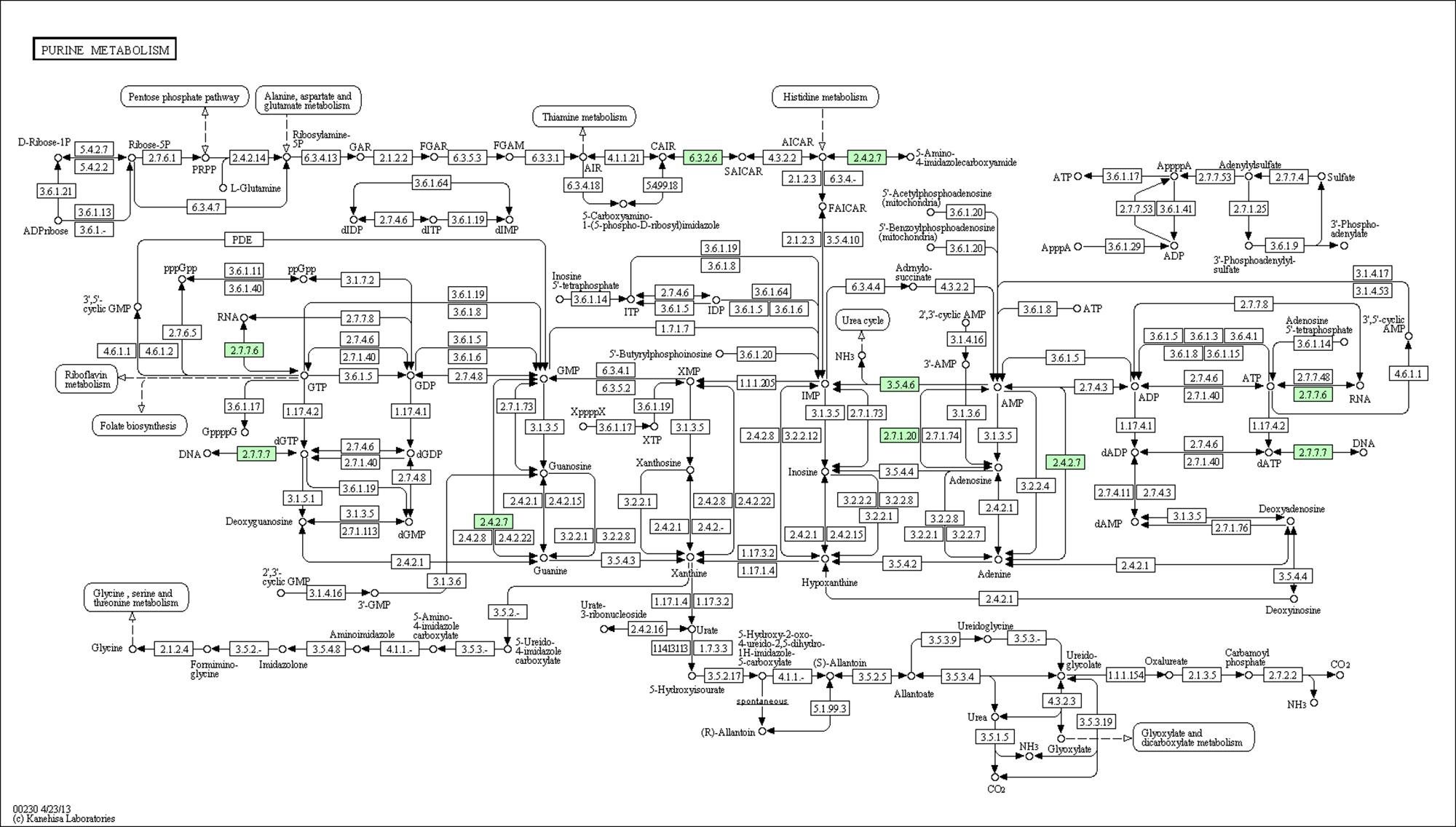

Supplement: Figure S8 — The locations of mutated genes in the purine Metabolism pathway. These frames that marketed with green color indicate the positions of each mutated gene. (TIF) [file pone.0074479.s009.tif]

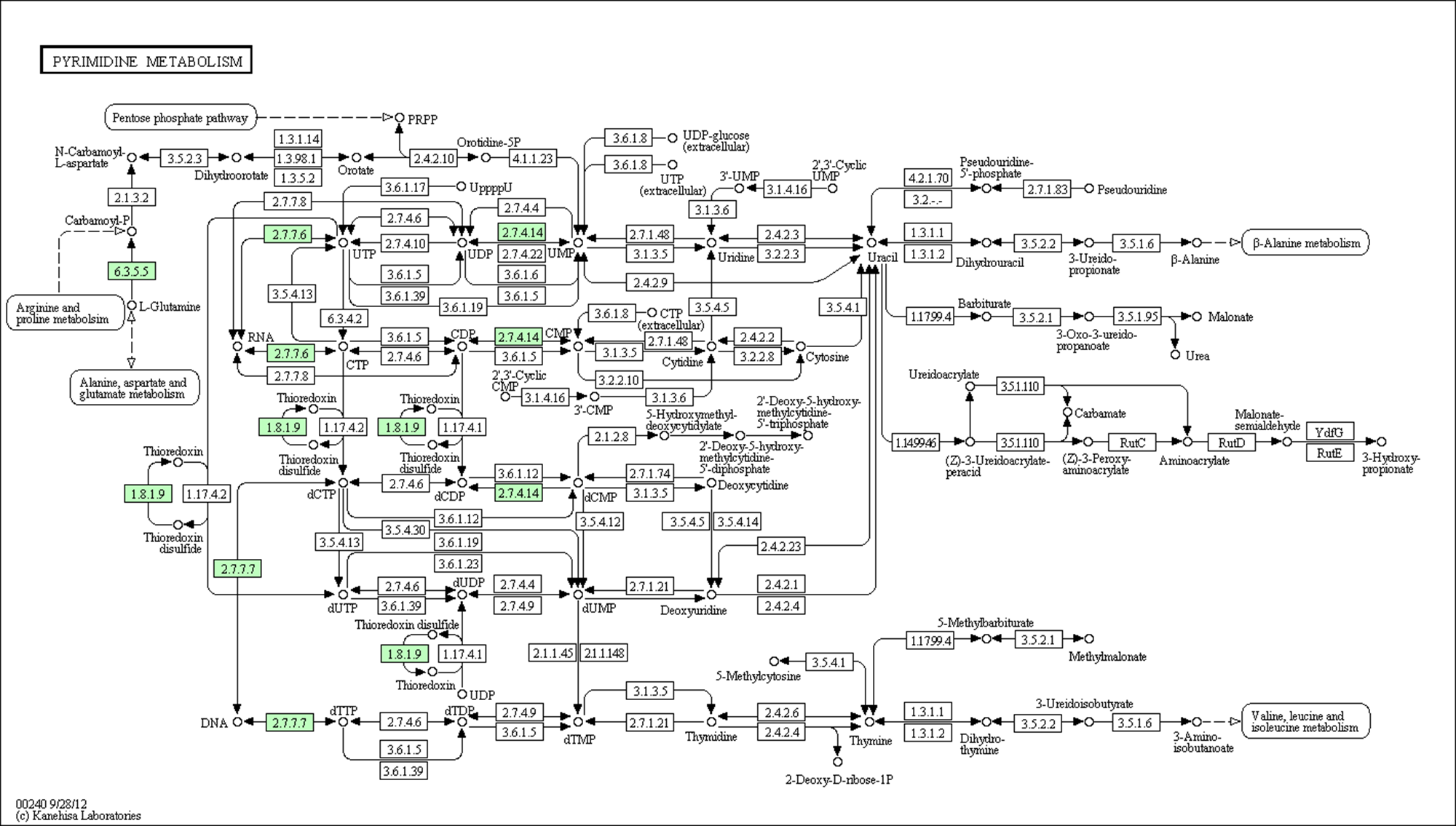

Supplement: Figure S9 — The locations of mutated genes in the pyrimidine Metabolism pathway. These frames that marketed with green color indicate the positions of each mutated gene. (TIF) [file pone.0074479.s010.tif]

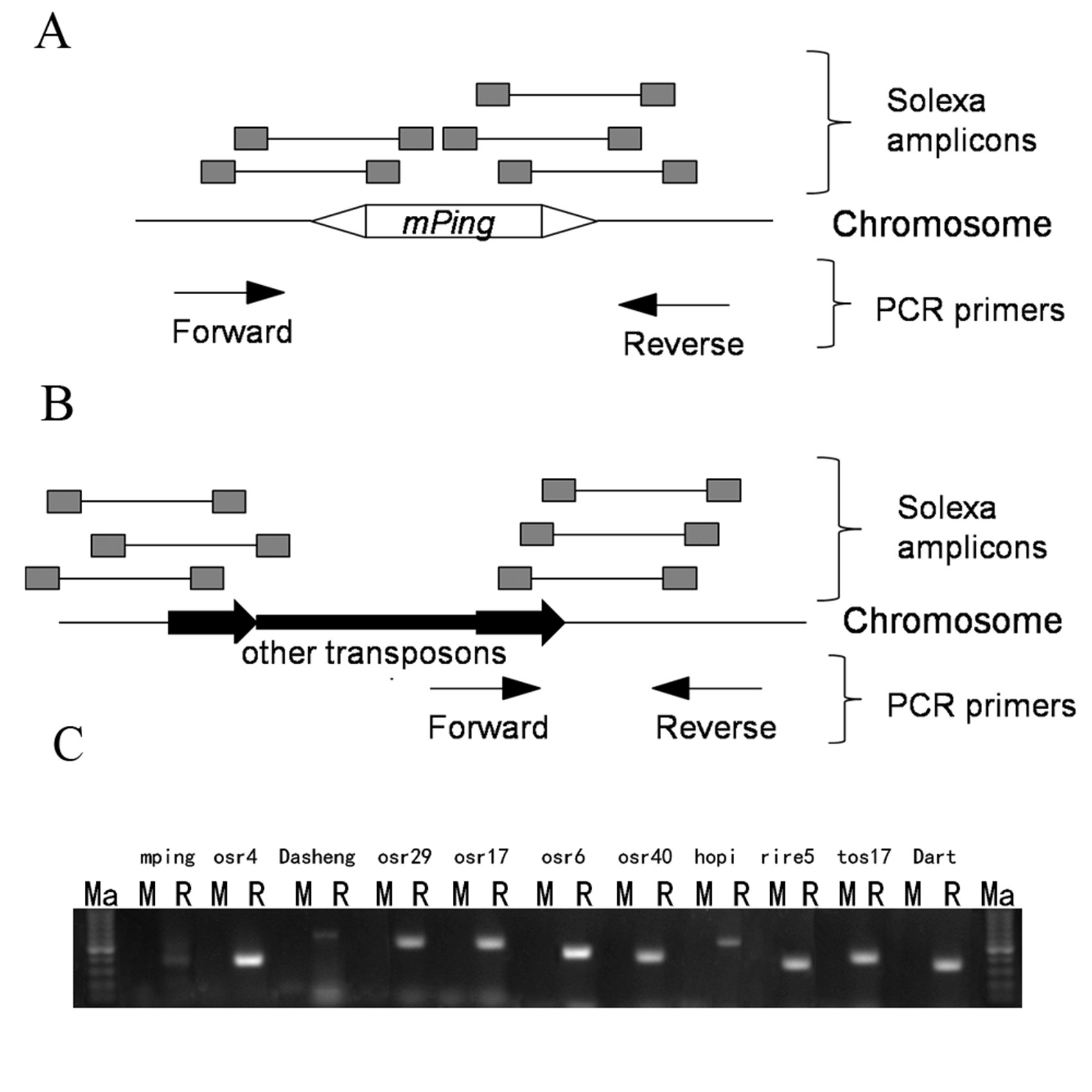

Supplement: Figure S10 — Identifications of transposable element (TE) insertions. The Illumina amplicons mapped are indicated above the genome sequence. (A) And (B) indicate the strategies of primer designing that is the same as Sabot et al. [42]. (C) Ma: represents Marker, M and R donate Matsumae and RZ35, respectively. (TIF) [file pone.0074479.s011.tif]
