# Supplementary material for: Genomewide Variation in an Introgression Line of Rice-Zizania Revealed by Whole-Genome re-Sequencing
Source: PLoS One. 2013 Sep 18;8(9):e74479. doi: 10.1371/journal.pone.0074479 (PMC3776793; doi:10.1371/journal.pone.0074479)
Supplement: Table S1 — The primer sequences for the identification of transposon elements insertion. (DOC) [file pone.0074479.s012.doc]

**Table S1.** The primer sequences for the identification of transposon elements insertion.

| Transposon | Chr. | Product Size | Sequence (5’-3’) |
| --- | --- | --- | --- |
| *osr4* | 1 | 456 | Forward: AAACCATAAAGTATTCTAAC |
|  |  |  | Reverse: CGGTGAATGAAACGAATC |
| *Dasheng* | 2 | 670 | Forward: TTGCTGGGATCATTTGTACC |
|  |  |  | Reverse: CAATCTGACCCTGTTTTTCCT |
| *osr6* | 4 | 550 | Forward: GTTGCCTCAAGAACAGTGCC |
|  |  |  | Reverse: GCGACCTCCGCATTCTC |
| *hopi* | 5 | 663 | Forward: CGTATTGGTTAAGGTTTCTATCT |
|  |  |  | Reverse: TTGCTTGTTCCGTGCC |
| *tos17* | 6 | 425 | Forward: AAGTTGCTGGGATCATTTGT |
|  |  |  | Reverse: CGAGTTGGCACTGTTCTTG |
| *mPing* | 6 | 430 | Forward: AAGCCTATCCTGCCAAGC |
|  |  |  | Reverse: GCCATGTCCTCCCAAAGA |
| *Dart* | 7 | 346 | Forward: CGGGAGAATGCGGAGGT |
|  |  |  | Reverse: CAACACTCAACCAGGGAGATTA |
| *rire5* | 7 | 390 | Forward: TTGCTGGGATCATTTGTACC |
|  |  |  | Reverse: GAATTTAGGCCCGATTGATT |
| *osr40* | 11 | 535 | Forward: GCCGTAAACCGTCCGCTA |
|  |  |  | Reverse: TGTCCAGGAATACATCACCGA |
| *osr17* | 11 | 630 | Forward: TCTCACTCCCTCTTTGTT |
|  |  |  | Reverse: ATTGTAGGACGTTTATCACT |
| *osr29* | 12 | 618 | Forward: CGGTTTCCTAAATCTACTT |
|  |  |  | Reverse: ATAATAATATCGGTGGCA |
