# Supplementary material for: Genomewide Variation in an Introgression Line of Rice-Zizania Revealed by Whole-Genome re-Sequencing
Source: PLoS One. 2013 Sep 18;8(9):e74479. doi: 10.1371/journal.pone.0074479 (PMC3776793; doi:10.1371/journal.pone.0074479)
Supplement: Table S2 — The primers sequences of anti-blast genes used in this study. (DOC) [file pone.0074479.s013.doc]

**Table S2.** The primers sequences of anti-blast genes used in this study.

| Gene-Name | Chr. | Product size | Sequence (5’-3’) |
| --- | --- | --- | --- |
| LOC_Os01g05620 | 1 | 225 | Forward: TCCTCTTGCCATCAAAG |
|  |  |  | Reverse: TAAGCGACAGCATCACG |
| LOC_Os01g10110 | 1 | 158 | Forward: CGCAACAAGTGGGACAGTAA |
|  |  |  | Reverse: GCCACCTCGCAGAAACC |
| LOC_Os01g57340 | 1 | 220 | Forward: AGGTTTCAAAGTTCCAGGGTT |
|  |  |  | Reverse: GAGGGAGGACGGCAGAT |
| LOC_Os04g32850 | 4 | 114 | Forward: CACCGAGGACAACGCCAACG |
|  |  |  | Reverse: GGAGGAGGAGATGAAATAGA |
| LOC_Os06g22460 | 6 | 354 | Forward: TGAACTTGTGAACCGATGT |
|  |  |  | Reverse: TCCTTTGGCAGCCTATC |
| LOC_Os06g29810 | 6 | 388 | Forward: AAAGTTTCAGCCATCCG |
|  |  |  | Reverse: TTACTCTTGCCATTTCCAC |
| LOC_Os09g32780 | 9 | 135 | Forward: TTGAGGGAATGACAGTTAGACA |
|  |  |  | Reverse: CGCCGCCCACATCGTAT |
| LOC_Os11g11790 | 11 | 301 | Forward: AGCAACGGAAGCGAAGG |
|  |  |  | Reverse: TGAGACCACGAAGTGATGTAAC |
| LOC_Os11g42010 | 11 | 424 | Forward: CATTGATGTTGAGGTGGAA |
|  |  |  | Reverse: TGACAGCCTGAAGAAGCA |
| LOC_Os12g18374 | 12 | 268 | Forward: GAGGCACCCAAGGTTGAAG |
|  |  |  | Reverse: CGCCGATGGCTGATACG |
