# Supplementary material for: Genomewide Variation in an Introgression Line of Rice-Zizania Revealed by Whole-Genome re-Sequencing
Source: PLoS One. 2013 Sep 18;8(9):e74479. doi: 10.1371/journal.pone.0074479 (PMC3776793; doi:10.1371/journal.pone.0074479)
Supplement: Table S3 — Information of these genes that new TE insertion was found within 1 kb. (DOC) [file pone.0074479.s014.doc]

**Table S3.** Information of these genes that new TE insertion was found within 1 kb

| **Chr.** | **Gene ID** | **Putative Function** | **Location of insertion** | **TE type** |
| --- | --- | --- | --- | --- |
| *1* | LOC_Os01g09384 | ankyrin repeat domain containing protein, putative, expressed | intron | *Osr4* |
| *1* | LOC_Os01g12830 | erythronate-4-phosphate dehydrogenase domain containing protein, expressed | intron | *Osr4* |
| *2* | LOC_Os02g22100 | OsRhmbd6 - Putative Rhomboid homologue, expressed | intron | *mPing* |
| *2* | LOC_Os02g51810 | expressed protein | upstream (-941 bp) | *mPing* |
| *3* | LOC_Os03g03100 | OsMADS50 - MADS-box family gene with MIKCc type-box, expressed | intron | *mPing* |
| *3* | LOC_Os03g03350 | polygalacturonase, putative, expressed | intron | *mPing* |
| *3* | LOC_Os03g21890 | potassium transporter, putative, expressed | upstream (-729 bp) | *mPing* |
| *3* | LOC_Os03g60870 | ras-related protein, putative, expressed | upstream (-896 bp) | *mPing* |
| *4* | LOC_Os04g54120 | serine/threonine-protein kinase receptor precursor, putative | intron | *Dasheng* |
| *4* | LOC_Os04g26920 | oxidoreductase, aldo/keto reductase family protein, putative, expressed | intron | *Osr6* |
| *5* | LOC_Os05g31254 | acetyltransferase, GNAT family, putative, expressed | intron | *Osr4* |
| *5* | LOC_Os05g11130 | cytochrome P450, putative, expressed | intron | *mPing* |
| *6* | LOC_Os06g44380 | ubiquitin carboxyl-terminal hydrolase family protein, expressed | intron | *Tos17* |
| *8* | LOC_Os08g17040 | retrotransposon protein, putative, unclassified | intron | *Osr29* |
| *8* | LOC_Os08g01110 | expressed protein | intron | *Dasheng* |
| *8* | LOC_Os08g08240 | transposon protein, putative, unclassified, expressed | intron | *mPing* |
| *9* | LOC_Os09g29740 | beta-expansin precursor, putative | upstream (-909 bp) | *Dasheng* |
| *11* | LOC_Os11g31950 | expressed protein | intron | *mPing* |
